# Compliance of pre-packaged food products with Nigerian food labelling guidelines: the NigeFE study

**DOI:** 10.3389/fnut.2025.1644344

**Published:** 2025-08-25

**Authors:** Gideon Onyedikachi Iheme, Ifeoma Mercy Egechizuorom, Linda Obianuju Edafioghor, Elizabeth Monday Okonkwo, Linda Eneh Olah, Barbara Adimchinobi Onyeonu, Oluwadamilare Emmanuel Kupoluyi, Hannah Chinyere Enuka, Adimchi Dike Adile, Idris Hajara, Obinna Chimela Ogbonna, Nwabuma Cynthia Asouzu, Chinyere Ikejiofor

**Affiliations:** ^1^Department of Food Studies, Nutrition and Dietetics, Uppsala University, Uppsala, Sweden; ^2^Department of Human Nutrition and Dietetics, Michael Okpara University of Agriculture, Umudike, Nigeria; ^3^Department of Nutrition and Dietetics, Federal Medical Centre Umuahia, Umuahia, Nigeria; ^4^Department of Nutrition and Dietetics, Federal Medical Centre Asaba, Asaba, Nigeria; ^5^Department of Nutrition and Dietetics, Jos University Teaching Hospital, Jos, Nigeria; ^6^Department of Human Nutrition and Dietetics, University of Ibadan, Ibadan, Nigeria; ^7^Department of Nutrition and Dietetics, National Rehabilitation Hospital, Dublin, Ireland; ^8^Department of Food and Nutrition Science, University of Reading, Reading, United Kingdom; ^9^Department of Nutrition and Dietetics, Federal Teaching Hospital, Gombe, Nigeria; ^10^Department of Pediatrics, Federal Teaching Hospital Katsina, Katsina, Nigeria; ^11^Department of Nutrition and Dietetics, Obafemi Awolowo University Teaching Hospital, Ile-Ife, Nigeria; ^12^Children’s Diabetes Team, East Kent Hospitals, University NHS Foundation Trust, Kent, United Kingdom; ^13^Nutrition Unit, National Agency for Food and Drug Administration and Control, Lagos, Nigeria

**Keywords:** food labelling, regulation, compliance, pre-packaged foods, Nigeria

## Abstract

**Background:**

Food labelling policy has become increasingly important in public health due to the rising burden of diet-related diseases. This study examines the compliance of pre-packaged foods sold in Nigerian markets with national food labelling guidelines.

**Methods:**

A total of 883 pre-packaged foods from broad categories were purposively enlisted from selected Nigerian stores and supermarkets. Food label information was evaluated against eleven (11) requirements stipulated by the National Agency for Food and Drug Administration and Control (NAFDAC) and components of the International Network for Food and Obesity/Non-communicable Diseases Research, Monitoring and Action Support (INFORMAS). Descriptive statistics and multiple linear regression were applied to assess compliance levels and their associated determinants.

**Results:**

The majority (>70.0%) of the pre-packaged foods complied with key labelling requirements except quantitative ingredient declaration (39.1%) as well as nutrient declaration of priority (56.5%), and non-priority (38.1%) nutrients. Overall, a mean food label compliance score of 9.19 + 1.43 out of a possible 11.0 was recorded. The inclusion of supplementary nutrition information and the declaration of non-priority nutrients in pre-packaged foods were each significantly (*p* < 0.01) associated with approximately a 0.50-point higher compliance score with national food labelling guidelines than pre-packaged foods lacking this information.

**Conclusion:**

High compliance with the guidelines was reported; however, the declarations of nutrient and quantitative ingredients were not sufficiently met. Prioritizing food labelling in policy formulation, alongside targeted support for food manufacturers particularly local producers will help address existing gaps across the food compliance spectrum.

## Introduction

Food labelling policy has been attracting attention in public health recently, due to the rising prevalence of mortality, disability, and cardiovascular diseases globally from diet-related causes (1). About 8 million deaths occur yearly from the consumption of unhealthy foods (2). Additionally, 38% of the global population was reported to be either obese or overweight (3), with developing countries bearing the most significant burden (4). Non-communicable diseases (NCDs) account for about 71% of all deaths, and three-quarters of these mortalities occur in developing countries (4).

The prevalence of obesity ranges from 20 to 35% in Nigeria (5, 6). The current food environment is a significant factor contributing to the surge in obesity prevalence, as it favours the sales of ultra-processed foods high in sugar, salt, and saturated fats (4, 7). In Brazil, the proportion of total calories from ultra-processed foods in household purchases rose from 20.8 to 25.4% between 2003 and subsequent years (8). However, between 2008–2009 and 2017–2018, the consumption of ultra-processed foods rose by 1.02 percentage points (9). Similarly, national evidence from the Global Diet Quality Project indicates that half (50%) of Nigerians consume unhealthy foods, predominantly pre-packaged foods, with 23 and 32% of the population reporting daily consumption of instant noodles and soft drinks, respectively (10).

The economic burden of these conditions has prompted food regulators to enact laws and guidelines that create a supportive food environment through policies encouraging healthy food choices among consumers and compelling food industries to enhance the nutritional quality of their food products. Evidence suggests that the implementation of recommended food environment policies was achieved by 11 countries (11). The most often prioritised policy issues were front-of-pack labelling, limiting the advertising of unhealthy foods, and taxing unhealthy foods. Chile has shown outstanding performance with food labelling and marketing laws that adhere to international best practices (12). A recent review of the global state of food labelling policies affirmed that 95 countries have mandatory nutrient declarations, with Africa accounting for approximately one-tenth of this total (nine countries) (13). While nutrition labelling continues to evolve from voluntary to mandatory with increasing advocacy and attention, there is a risk that this transition may obscure evidence on compliance with longstanding mandatory general food labelling requirements.

The CODEX Alimentarius periodically publishes and updates international recommendations and guidelines on the core information to be captured on the labels of pre-packaged products (14). This guideline has been well-adopted globally as several countries and regions both developed; USA (15), Europe (16), United Kingdom (17), Canada (18) and developing; Brazil (19) South Africa (20), Ghana (21, 22) have outlined national or regional mandatory labelling regulations for pre-packaged foods. In Nigeria, according to the National Agency for Food and Drugs Administration and Control (NAFDAC), pre-packaged foods should contain specific elements that guide consumers in making healthy dietary choices, presented in clear, legible, and bold formats. NAFDAC Act specifies that no one may manufacture, import, distribute, advertise, or display pre-packaged foods for sale unless they have met the proper labelling standards (23). The National Guide to Food Labelling, developed by NAFDAC (24), outlines 11 food labelling requirements that closely conform to the international Codex recommendations and guidelines (14). Despite the efforts made by regulatory bodies to improve consumers’ access to food label information, there appears to be a lack of substantial evidence to demonstrate the extent of disclosure of these general food label requirements. Only a few studies have focused on online product labelling (25), addressed limited food label components (26), or relied on small sample sizes (27, 28). On the other hand, multiple sources of pooled evidence highlight an established discourse around the nutrition labelling landscape, including nutrition/health claims (29–34).

Given the disparity in the weight of evidence on food and nutrition labelling, this study aims to comprehensively assess the extent and determinants of compliance with food labelling guidelines among pre-packaged foods in Nigeria.

## Methods

### Study design

This study is part of the larger NigeFE (Nigerian Food Environment) study. We employed a descriptive, cross-sectional, quantitative content analysis to collate and audit information on pre-packaged foods, following NAFDAC guidelines and INFORMAS (International Network for Food and Obesity/non-communicable Disease Research, Monitoring and Action Support) protocols.

### Food label analysis and information repository (FLAIR database)

The FLAIR database is Nigeria’s repository for pre-packaged food and beverages. This study reports findings of the initial or preliminary FLAIR database, which contains 883 foods and is expected to expand considerably over time.

FLAIR Database 2022 and 2023 Preliminary Data Collection: Between October 2022 and August 2023, data collection was conducted in selected major closed markets (supermarkets, stalls) in the capital cities of nine Nigerian states: Abakaliki, Abeokuta, Asaba, Enugu, Jos, Katsina, Gombe, Osogbo, and Umuahia. Data entry sheets were created in a smartphone application, with enabled access to cameras to capture clear images of all sides of branded labels. As all information was collated into the same repository, products of the same or different sizes duplicated in other retail outlets or regions were captured only once. However, products that appear similar but possess distinct organoleptic attributes were captured. For each food category, brands or products with national or regional popularity and visibility were selected for inclusion. All information related to food labels was coded into a database using a mobile application. We used the national food labelling requirements and the INFORMAS food label components to determine the necessary information (24, 35).

### Variables and outcome

All data collected were transferred to Excel for pre-processing before being exported to IBM SPSS for further categorisation and formal analysis.

### Food categories

The foods were categorised following the classification provided by the Global Food Monitoring Group. Global food Monitoring protocol classified foods into these broad divisions: Confectionary, bread, and bakery products; cereal, and cereal products; dairy, edible oils and oil emulsions; fruit and vegetables, sauces and spreads, snack foods, processed fish; unique foods, meat and meat products, non-alcoholic beverages and alcohol. New categories were added for food products or their groups which did not fit into the existing categories.

### National guidelines

The National Guide to Food Labelling, developed by NAFDAC, outlined 11 food labelling requirements that products are expected to adhere to (24). This closely conforms to the international Codex recommendations and guidelines (14). This 11 mandatory information comprise; declaration of information on the food label, name/description of food, list/statement of ingredients, quantitative ingredient declaration (QUID), net quantity, date of minimum durability, special storage instruction or conditions of use, batch number, name and address, instructions for use, allergen declaration.

Although NAFDAC has published new regulations (36), some of these details are generic and cover broad aspects such as offences and penalties. The national guide to food labelling provides clear-cut and well-defined requirements for compliance.

### INFORMAS food labelling components

The INFORMAS protocol outlines key information needed in food labelling assessment studies (35). This includes a list of ingredients, nutrient declarations, and supplementary nutrition information that have been mentioned as the central/core components of the food label (35).

### Data analysis

A product that met or was not affected by each specific component of the food label requirement was assigned a score of 1. In instances where a component was only partially met, a score of 0.5 was assigned. Therefore, a food product that fulfilled all 11 labelling requirements attained a maximum compliance score of 11 points.

Descriptive statistics (frequency, percentage, mean and standard deviation) were computed for the continuous and categorical variables. Crosstabulation was used to describe the distribution of food label compliance scores across food categories and place of manufacture.

Multiple regression analysis was used to examine the association between food label compliance scores and key characteristics of food and nutrition labelling. The binary variables indicating whether a product was foreign-manufactured/imported, belonged to a healthy food category, declared priority nutrients, declared non-priority nutrients, and included supplementary nutrition information were included in the model as predictors, adjusted to control for their mutual influence. Also, food label compliance scores formed the continuous dependent variable.

The model met the acceptable threshold (<5) for multicollinearity, as determined by the Variance Inflation Factor. Assumptions of linearity, homoscedasticity, and normality of residuals were satisfied, as verified through residual plots and a non-significant Shapiro–Wilk test (*p* > 0.05). Significance was judged at *p* < 0.05. All analyses were done using SPSS Version 28.

## Results

### Compliance with food labelling requirements/components across local and foreign products

A total of 883 pre-packaged foods were surveyed. Table 1 shows the compliance of locally made and imported pre-packaged food products with food labelling requirements. The finding reported a high degree of compliance with food labelling regulations.

**Table 1:** Compliance with food labelling requirements/components across local and foreign products.

Variables	ALL *N* (%)	Local *N* (%)	Foreign *N* (%)
NAFDAC food labelling requirement			
Declaration of information on food label	785 (88.9)	384 (89.5)	401 (88.3)
Name or description of food	876 (99.2)	426 (99.3)	450 (99.1)
List/statement of ingredients	815 (92.3)	396 (92.3)	419 (92.3)
Quantitative ingredient declaration (QUID)	345 (39.1)	132 (30.8)	213 (46.9)
Net quantity	835 (94.6)	395 (92.1)	440 (96.9)
Date of minimum durability	776 (87.9)	376 (87.6)	400 (88.1)
Special storage instructions or conditions of use	660 (74.7)	358 (83.4)	302 (66.5)
Batch number	778 (88.1)	373 (86.9)	405 (89.2)
Name and address of manufacture	848 (96.0)	425 (99.1)	423 (93.2)
Instructions for use	635 (71.9)	331 (77.2)	304 (67.0)
Allergen declaration	715 (81.0)	300 (69.9)	415 (91.4)
Mean compliance	9.19 ± 1.43	9.15 ± 9.50	9.22 ± 1.37
INFORMAS food label components			
Ingredient list	815 (92.3)	396 (92.3)	419 (92.3)
QUID priority ingredients	127 (14.5)	47 (11.1)	80 (17.7)
QUID non-priority ingredients	187 (21.3)	82 (19.3)	105 (23.2)
Nutrient declaration for priority nutrients	499 (56.5)	249 (58.0)	250 (55.1)
Nutrient declaration for non-priority nutrients	336 (38.1)	157 (36.6)	179 (39.4)

Findings revealed that majority of the pre-packaged products reported the following information; declaration of food label information (88.9%), product name and description (99.2%), list/statement of ingredients (92.7%), net quantity (94.6%), date of minimum durability (87.9%), batch number (88.1%) and manufacturer’s details (96.0%). The variation between local and foreign products for these attributes had a difference of no more than ±5%.

Wide variations were observed in other requirements as local food packages had higher special storage instructions (local: 83.4%, foreign: 66.5%), instructions for use (local: 77.2%, foreign: 67%) but lower allergen declarations (local: 69.9% foreign: 91.4%) than their foreign counterparts. Quantitative ingredient declaration was the least complied (39.1%) food labelling requirement, with foreign products (46.9%) having a higher compliance rate than local (30.8%) pre-packaged food products. The overall mean compliance with national food label requirements was 9.18 ± 1.43 out of a possible 11 points. Pre-packaged food products manufactured outside Nigeria recorded slightly higher compliance with national guidelines than local pre-packaged foods (Local: 9.15 ± 9.50; Foreign: 9.22 ± 1.37).

According to the INFORMAS guidelines, the majority of local (98.6%) and foreign (95.6%) pre-packaged foods provided information on the ingredient lists. More than half (56.5%) of pre-packaged food products declared information on priority nutrients, with comparatively higher adherence observed in local products (local: 58.1%; foreign: 55.1%). The non-priority nutrient declaration had an overall lower reportage (38.1%), which was slightly higher for foreign products (39.4%) than their domestic counterparts (39.4%). While compliance with QUID was generally low, higher reporting was observed in non-priority QUID (21.3%) than in priority QUID (14.3%), with foreign (17.7–23.2%) pre-packaged foods recording the highest compliance in both categories compared to local (11.1–19.3%) products.

### Food category distribution of compliance with food labelling requirements/components

Information on pre-packaged food compliance across food categories is summarised in Tables 2, 3. The majority (>80%) of all food groups adhered to the declaration of information on the food label, including the name or description of the food, list or statement of ingredients, net quantity, name and address of the manufacturer, and instructions for use. Adherence to batch number and allergen declaration reporting was found in at least 50% of all food categories.

**Table 2:** Food category distribution of compliance with NAFDAC food labelling requirements/components.

Food category	Declaration Info	Name/description	List of ingredients	QUID	Net Quantity	Durability date	Storage info	Batch number	Manufacturer info	Instructions of use	Allergen declaration
Confectionary	100	100	95.7	31.5	80.4	72.8	65.2	72.8	95.7	97.8	53.3
Bread and Bakery	100	100	96.7	20.8	97.9	97.9	96.9	95.8	99	62.5	87.5
Cereals and products	95.9	98.6	91.8	20.5	100	97.3	94.5	94.5	98.6	91.8	78.1
Dairy and products	93.4	97.8	98.9	57.1	83.5	95.6	94.5	94.5	89	95.6	95.6
Edible oils	100	95.5	86.4	63.6	100	68.2	90.9	77.3	90.9	50	86.4
Fruits and vegetables	97.6	100	85	64.4	100	96.1	92.1	92.1	91.3	86.6	85.8
Sauces and spreads	100	95.2	100	100	100	100	100	100	100	0	85.7
Snack foods	100	100	84.4	3.1	90.6	100	93.8	100	96.9	100	50
Processed fish	100	100	91.7	25	100	100	100	100	100	100	50
Special foods	95	95	100	85	100	100	100	100	100	95	85
Meat and meat products	100	100	100	33.3	100	100	100	100	100	100	66.7
Non-alcoholic beverages	89.3	100	98.1	22.3	96.1	96.1	76.7	63.1	100	40.8	66
Alcohol	55.7	99.4	89.8	37.1	96.4	96.4	15.6	91.6	97	46.7	100
Processed tuber products	100	100	95.8	20.8	100	100	100	100	100	100	66.7
Total	88.9	99.2	92.3	39.1	94.6	94.6	74.7	88.1	96	71.9	81

Color codes indicate relative reportage levels based on data distribution — Green: Very High, Yellow: High, Orange: Moderate, Red: Low.

**Table 3:** Food category distribution of compliance with INFORMAS food labelling requirements/components.

Food category	Ingredient list	Priority ingredients	Non-priority ingredients	Priority nutrient	Non priority nutrient	Supplementary nutrition
Confectionary	95.7	19.6	9.8	58.7	41.8	67.4
Bread and Bakery	96.7	7.8	22.2	74.4	55.6	82.3
Cereals and products	91.8	4.2	16.7	100	58.9	100
Dairy and products	98.9	6.6	38.5	95.6	84.6	58.2
Edible oils	86.4	0	90.9	90.9	45.5	81.8
Fruits and vegetables	85	17.3	32.3	33.9	15	67.7
Sauces and spreads	100	100	100	33.3	33.3	33.3
Snack foods	84.4	0	3.1	78.1	50	78.1
Processed fish	91.7	8.3	8.3	16.7	16.7	100
Special foods	100	10.5	42.1	89.5	94.7	35
Meat and meat products	100	0	0	0	33.3	33.3
Non-alcoholic beverages	98.1	17.5	6.8	76.7	36.9	89.3
Alcohol	89.8	17.4	2.4	3	3	12.6
Processed tuber products	95.8	0	33.3	83.3	50	87.5
Total	92.7	14.5	21.4	57	38.4	63.1

Color codes indicate relative reportage levels based on data distribution — Green: Very High, Yellow: High, Orange: Moderate, Red: Low.

Except sauces and spreads, and other food categories (diary, edible oils and oil emulsions, fruits and vegetables, special foods) where all and half of the products quantitatively declared their ingredients, respectively, QUID was reported in about one in five of all products within the bread and bakery (20.8%), cereal and cereal products (20.5%), processed fish (25%), non-alcoholic beverages (23.3%), and processed tuber products (20.8%) categories.

According to the INFORMAS guideline, the majority (>80%) of all the food categories displayed the list of ingredients in their products. All (100%) sauce and spread products declared the number of priority ingredients, compared to less than 20% of other food groups that provided information on the quantity of priority ingredients. Similarly, all (100%) and the majority (90.0%) of sauce and spread products and edible oils/oil emulsions declared the quantity of non-priority ingredients, respectively. Low or negligible compliance with quantity declaration of non-priority ingredients was reported in meat and meat products (0.0%), alcohol products (2.4%), and snack foods (3.1%).

Bulk (>70%) of all the products from the various food categories complied with the nutrient declaration for priority nutrients except alcohol products (3.0%), meat and meat products (0.0%), processed fish (16.7%), sauce and spread products (33.3%), fruits and vegetable products (33.9%) and confectionery (58.7%). Compliance with nutrient declarations for non-priority nutrients was highest among special foods (94.7%) and dairy products (58.9%), with alcohol products (3%), processed fish products (15.0%), and fruit and vegetable products (16.7%) recording the least compliance.

Supplementary nutrition information was captured in the majority (>70%) of processed tuber products, non-alcoholic beverages (powdered and liquid), processed fish, snack foods, cereal and cereal products and bread and bakery products. In comparison, a lower proportion of pre-packaged food within the alcohol (12.6%) and special foods (35.0%) categories included supplementary nutrition information.

### Factors influencing compliance with food label requirements

Information on the determinants of compliance with food label requirements is presented in Table 4. A significant association (*p* < 0.01) was found between the declaration of non-priority nutrients and supplementary nutrition information, as required by food label regulations. Specifically, the declaration of non-priority nutrients was associated with a 0.51-point higher compliance score than not declaring non-priority nutrients. Similarly, the inclusion or presence of supplementary nutrition information was associated with a 0.50-point elevation in compliance score compared to its absence.

**Table 4:** Factors influencing compliance with food label requirements.

Variables	*B*	Std Error	*T*	Sig	95% CI
Constant	6.91	0.25	27.56	<0.001	6.42, 7.40
Country of manufacture—foreign products	0.10	0.09	1.08	0.28	−0.08, 0.28
Healthy food category	0.07	0.09	0.85	0.40	−0.10, 0.24
Nutrient declaration for priority nutrients	0.26	0.13	1.93	0.05	−0.004, 0.52
Nutrient declaration for non-priority nutrients	0.51	0.12	4.35	<0.001	0.28, 0.75
Supplementary nutrition information	0.50	0.12	4.26	<0.001	0.27, 0.73

Dependent variable: food label compliance score.

## Discussion

Food labels are essential for encouraging healthy eating habits, supporting international initiatives to improve food safety, empowering consumers with the correct information to make healthier food decisions, and ultimately reducing the burden of NCDs.

The study findings indicate an overall high level of compliance with national food labelling regulations, which consequently aligns with Codex Alimentarius mandatory guidelines for food product declarations (14). This conformity positions Nigeria’s pre-packaged food market for enhanced cross-border trade opportunities and greater integration into international markets.

Specifically, the results revealed that nearly all (>90%) pre-packaged foods disclosed the product names, list of ingredients and manufacturer’s contact information. This compares closely with previous studies, which reported full (100%) compliance with the disclosure of the name and ingredient list, as well as the inclusion of manufacturer contact information on the majority of products (27, 37, 38). Regulatory agencies mandate the accurate disclosure of this information to ensure transparency, facilitate brand identification and support product traceability.

Conversely, instructions for use and, in particular, QUID exhibited the lowest levels of compliance. While disclosure of the instruction for use showed relatively modest adherence, it was notably higher than the rates reported in previous studies (27, 37, 39) but lower in another study conducted in India (40). Instructions for use statements are required to provide consumers with precise instructions on the safe preparation, handling or use of food products. Although QUID is considered a global best practice for food labelling, it remains an optional disclosure internationally, as captured in the CODEX standard for labelling of pre-packaged foods (14) and other developed countries (15, 18), including Nigeria (24). QUID should only be mandatorily listed when a product name or nutrition/health claims highlight the presence of a specific ingredient (14, 24). These exemptions may partly explain the low QUID compliance observed in this study. Thus, suggesting that food manufacturers employ deceptive marketing techniques to project foods as containing healthful ingredients without providing quantitative substantiation.

When stratified across place of manufacture, a notable compliance gap between locally produced and imported products was observed, with locally produced items exhibiting lower compliance rates. This trend contrasts with previous studies, such as one conducted in Libya, where local foods demonstrated greater conformity with Libyan food labelling regulations than imported foods (28). While local products excelled in providing usage and storage directions, foreign items exhibit higher compliance in other areas, such as allergy and QUID disclosure. These results slightly corroborate those of a previous study, which found that foreign products exhibited higher compliance in areas such as the list of ingredients, storage instructions, net content, and directions for use (37). Similarly, another study also reported the presence of less stringent allergen regulation and compliance in developing countries (41). The disparity in this study underscores the urgent need for more funding for food labelling education and awareness campaigns, with a focus on local food producers. Improving allergen and QUID disclosure is critical for preventing health risks and building consumer trust (42). Furthermore, it enhances the economic potential of local products by increasing their competitiveness in international trade and allowing tourists to enjoy indigenous foods with confidence in their safety (43, 44).

Although overall QUID disclosure was low, snack foods, followed by cereal and cereal products, had the least QUID disclosure, while edible oils demonstrated relatively high compliance. Thus, while snacks and cereal products are associated with nutrition and health claims in literature (45–47), this present study has shown a lack of transparent quantification of these purported beneficial ingredients. Edible oils are predominantly derived from oil seeds such as palm, soybean, rapeseed (canola), sunflower, peanut, and olive fruits (48), which are usually referenced in the products descriptive name (48), and therefore are expected to declare these ingredients quantitatively (14, 24). Furthermore, the oxidative sensitivity of edible oils is mainly dependent on light and temperature and this affects their shelf-life (49, 50), thus explaining variability in the low and high adherence with date of minimum durability and storage information, respectively. Similar to edible oils, sauces, and spreads, alcoholic and non-alcoholic beverages had low compliance with instructions for use, likely because their application is self-explanatory and straightforward (36). The low compliance of alcoholic beverages with not only instructions for use, as previously highlighted, but also storage information underscores the prioritization by food manufacturers on other core labelling elements specific to these products, such as ingredient list, alcohol by volume and allergen declaration (36, 51). Similarly, international perspectives also affirmed that storage information is often omitted from alcohol labels unless directly related to safety concerns or post-opening refrigeration needs (52).

Our study reports that the INFORMAS food labelling components compare closely with findings from Franco-Arellano et al. (43), who observed that food products of all categories tend to have higher compliance with ingredient lists and priority nutrient declarations, but this considerably declines as quantitative and non-priority elements of these nutrients are further investigated. Supported by Steele-Dadzie et al. (37), all categories of products declared at least the “big four” nutrients, but this dropped when the “big eight” (priority) nutrients were examined, and even further when the “big eight nutrients” (non-priority) information was reviewed. The widespread inclusion of supplementary nutrition information in pre-packaged products, when compared to nutrient declarations, does not comply with Codex regulations that supplementary food labels should be optional and should only be provided to complement, rather than replace, nutrient declarations (14). Although the literacy rate in Nigeria is valued at 63% (44), the fact that these national statistics mask regional gaps makes a case for exemptions to support the primary use of supplementary nutrition information in populations or regions with lower literacy levels.

Nutrition labelling encompasses information used to educate consumers about a product’s nutritional qualities. Its two main components are supplementary nutrition information and the nutrient declaration. From this study, factors such as the declaration of non-priority nutrients and supplementary nutrition were found to have a significant association with food label requirements. Non-priority nutrients are occasionally listed on labels to give consumers more clarity on the item’s overall nutritional profile. Additional labelling requirements could be necessary for supplementary details, such as claims of extra vitamins, minerals, or dietary fibre, to guarantee that consumers can base their decisions on reliable, consistent information. This often impacts rules, as authorities may demand that any additional claims be made explicit and supported by facts.

Generally, food labels have shown great promise in terms of compliance with outlined regulatory guidelines; however, aspects of nutrient and ingredient quantification still fall short. This may be attributed to the recent regulatory transition from a claims-associated mandatory declaration to a mandatory nutrient declaration of all branded food products in Nigeria, which may need some time to take full effect (24, 36). On the other hand, the recent global food labelling policy mapping, which revealed the paucity of food labelling policies in Africa when compared to other regions (13), has demonstrated that while government regulations and standards enforce the implementation of health-related food and nutrition policies (53), food label regulations will receive sufficient and sustained government priority when they are explicitly captured within the policy targets (54).

## Limitations of the study

While this study is the first to provide a comprehensive audit of pre-packaged food product labels to assess compliance with national food labelling guidelines, several limitations are acknowledged.

First, the preliminary nature of the study limited the scope of sampling, restricting robust regional analysis or state-level analyses that could reveal location-specific enforcement patterns. Consequently, the findings may lack the robustness required to provide comprehensive explanations of enforcement variations, potentially skewing interpretations of the data.

Also, sole reliance on the information presented on product labels prevented direct engagement with manufacturers, which could have provided deeper insights into the reasons behind compliance or non-compliance with specific labelling requirements. This lack of verification may have influenced how products were rated.

## Conclusion

Food labels are essential tools promoting healthier eating habits, ensuring food safety and addressing the rising burden of non-communicable diseases. While food labelling is mandatory in most countries, compliance varies, especially between local and imported products as well as across food categories.

The study highlights an overall high compliance with food labelling guidelines, but notable gaps in compliance for quantitative ingredient declaration (QUID), usage instructions, storage information, allergen and nutrient declarations. Furthermore, disclosure of nutrition information was significantly associated with higher overall labelling compliance.

Improving compliance particularly among local producers requires targeted regulatory enforcement and capacity-building through specialized training and education on both food and nutrition labelling. In addition, shifting from generalized labelling requirements to product-specific regulations would help identify relevant labelling elements and allow for justified exemptions where appropriate.

Finally, integrating food labelling into broader national food and nutrition policies, alongside expanded research on the impact of labelling interventions for both industry and consumers, will strengthen the evidence base and regulatory capacity needed to drive sustained improvements.

## Data Availability

The raw data supporting the conclusions of this article will be made available by the authors without undue reservation.
